# Effect of Initial State and Deformation Conditions on the Hot Deformation Behavior of M50NiL Steel

**DOI:** 10.3390/ma13235367

**Published:** 2020-11-26

**Authors:** Yan Zhang, Ming Yang, Shaolei Long, Bo Li, Yilong Liang, Shaowei Ma

**Affiliations:** 1 College of Materials and Metallurgy, Guizhou University, Guiyang 550025, China; zhang01718@163.com (Y.Z.); liangyilong@126.com (Y.L.); 2 Guizhou Key Laboratory for Mechanical Behavior and Microstructure of Materials, Guiyang 550025, China; 3 National & Local Joint Engineering Laboratory for High-performance Metal Structure Material and Advanced Manufacturing Technology, Guiyang 550025, China; 4 Guizhou Electric Power Research Institute, Guiyang 550007, China; zy15508512961@163.com; 5 Guizhou Institute of Technology, College of Materials and Energy Engineering, Guiyang 550025, China; 6 National Interligent Foundry Innovation Center, Yinchuan 750021, China; msw2654665822@163.com

**Keywords:** M50NiL steel, critical strain, dynamic recrystallization, initial grain size, second phase

## Abstract

M50NiL steel, which belongs to a new generation of case-hardening steels used in aerospace bearing applications, is used mainly for the manufacturing of aerospace transmission components that operate under high temperatures. In this study, the effects of the hot deformation parameters and the initial microstructure on the hot deformation behavior of M50NiL steel were investigated through Gleeble-3500 isothermal hot compression tests. The experimental results demonstrated that the critical stain of dynamic recrystallization and the deformation activation energy of the coarse-grained samples were higher than those of the fine-grained samples. This is attributed to the difficulty of deformation and the dynamic recrystallization behavior of coarse-grained samples. Moreover, fine-grained samples contain a large number of dispersed phases, which can pin the grain boundaries and inhibit the growth of recrystallized grains. Such phenomena are beneficial for obtaining finer and more uniform microstructures in M50NiL steel. The experimental results can provide a useful reference for preparing M50NiL steel with excellent mechanical properties.

## 1. Introduction

In future advanced gas turbine engines, the main shaft bearing may operate under extremely high temperature conditions, high loads, and harsh corrosive environments [1]. In order to satisfy the requirements concerning bearing materials, aircraft engine bearings should combine the characteristics of good corrosion resistance and high surface hardness for wear resistance while maintaining a core with good ductility, as well as fracture and impact toughness. M50NiL steel, which belongs to a new generation of case-hardening steels, has been developed by improving the M50 bearing steel. More specifically, M50NiL steel is a secondary hardening steel with a low carbon content and high contents of Cr, Mo, Ni, and V. Therefore, M50NiL steel has a composite microstructure, which, after surface treatment, obtains a case-hardened surface and a relatively tough core, meeting the requirements for aircraft engine components that work under harsh conditions [2,3]. The manufacturing processes of bearing components made from M50NiL steel include hot ring rolling, heat treatment, and machining. Among them, hot ring rolling has considerable effects on the microstructure and mechanical properties. Therefore, investigating the hot deformation behavior of M50NiL steel is of great importance in the production of bearing components with excellent microstructure and mechanical properties.

Hot deformation parameters such as strain, strain rate, and deformation temperature are crucial for controlling the microstructure of the final products. Potential abnormal deformation conditions can induce surface cracks or inhomogeneous grain size distribution [4]. In general, work hardening, dynamic recovery, and dynamic recrystallization occur during hot deformation. Dynamic recrystallization can lead to significant refinement of the microstructure, which can improve the mechanical properties and reduce the hot deformation resistance [5]. Consequently, it is vital to investigate the relationship between deformation parameters and dynamic recrystallization behavior (DRX). Several studies [6,7,8] have investigated the effect of hot deformation parameters on the DRX of alloys. However, results concerning M50NiL steel have rarely been reported.

Apart from the hot deformation parameters, the initial state conditions, such as grain size and second phase particles, also have an effect on DRX; however, they are often neglected. A few studies [9,10] focusing on the effect of initial grain size have been conducted to determine the relationship between steady-state grain size and initial grain size. The most common observations among these studies are that the steady-state grain size depends more on the steady-state conditions than the initial grain size, and that the dynamic recrystallization kinetics decrease with an increase in the initial grain size [11]. In addition, some studies have indicated that second-phase particles can either accelerate or suppress recrystallization depending on their size, that coarse particles are able to stimulate the nucleation of dynamic recrystallization grains, and that fine second-phase particles can hinder recrystallization as they can suppress the growth of dynamic recrystallization grains [12,13,14,15]. Hence, it is important to investigate the effect of the initial state and deformation parameters on the hot deformation behavior of M50NiL steel. Ji et al. [16] studied the hot deformation behavior of M50NiL steel and proposed a strain-compensated Arrhenius equation and a modified Field–Backofen equation for M50NiL steel. However, most studies on M50NiL steel have focused on surface modifications. For example, Yan et al. [17] investigated the depth-related microstructure and wear properties of the rare earth nitrocarburized layer of M50NiL steel, and Li et al. [18] analyzed the tribological properties and wear mechanisms of as-deposited films on M50NiL steel.

In this paper, the effect of the initial microstructure and hot deformation conditions on the hot deformation behavior of M50NiL steel was investigated to optimize its microstructure and mechanical properties. Different methods, including true stress–strain curves, a critical strain model, and microstructural characterization, were employed to analyze the effect of the initial microstructure and hot deformation conditions on the hot deformation behavior of M50NiL steel. The results can provide a valuable reference for obtaining M50NiL steel with excellent mechanical properties and an appropriate microstructure.

## 2. Materials and Methods

### 2.1. Materials

**Figure 1:**
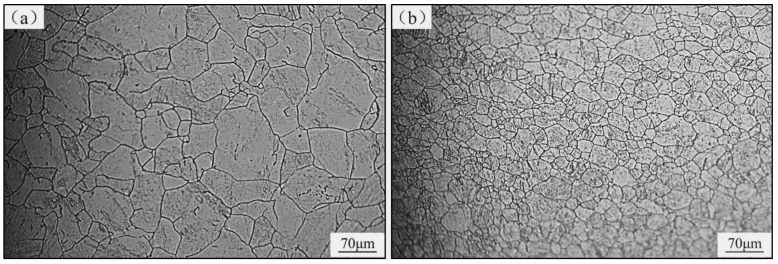
Optical micrographs of the M50NiL steel samples with coarse and fine grains prior to deformation: (**a**) coarse-grained sample (obtained by heating at 1150 °C for 30 min); (**b**) fine-grained sample (obtained by heating at 1100 °C for 5 min).

**Figure 2:**
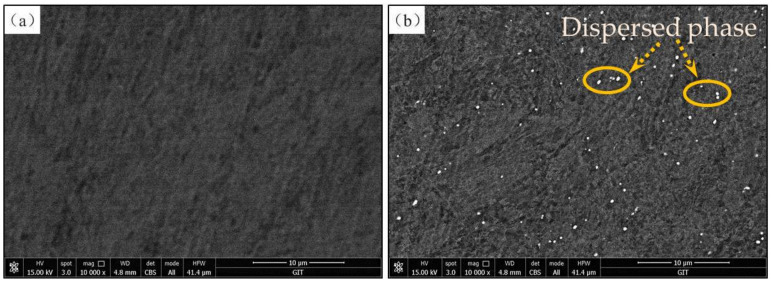
Scanning electron microscopy equipped with a backscattered electron (SEM–BSE) of the M50NiL steel samples with coarse and fine grains prior to deformation: (**a**) coarse-grained sample; (**b**) fine-grained sample.

**Figure 3:**
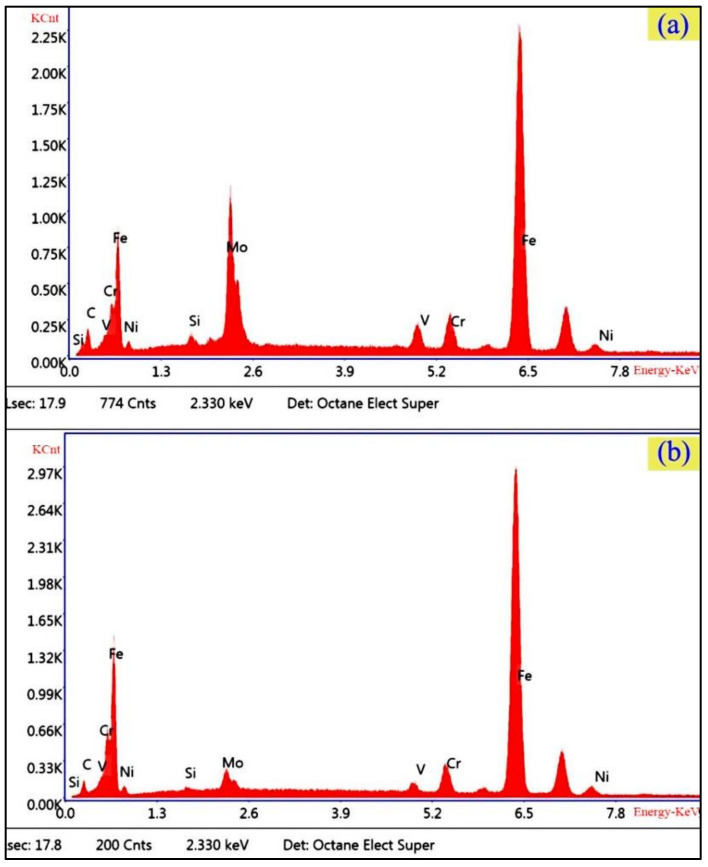
Energy dispersive X-ray spectroscopy (EDS) results of (**a**) the dispersed phase and (**b**) the matrix of M50NiL steel.

In this work, the annealed M50NiL steel (provided by Baoshan Iron & Steel Co., Ltd. in china) was used to investigate the effect of the initial state and deformation conditions on hot deformation behavior. Its chemical composition is given in Table 1. According to [19], when M50NiL is heated to 1100 °C or above, carbides dissolve; thus, the grain boundary is not able to be pinned by carbides and the grains grow abnormally as a result. Therefore, specimens were heated at 1100 °C for 5 min and at 1150 °C for 30 min in order to obtain two different initial states for studying the effect of different grain sizes and microchemistry on the hot deformation behavior of M50NiL steel. As seen in Figure 1a,b, equiaxed grains with a size of 69.11 μm and 23.95 μm, respectively, were obtained after the different heat treatments. Moreover, a portion of the fine-grained specimen exhibited the fine spheroidal dispersed phase, the size of which was measured as 0.27–0.32 μm (Figure 2b). The presence of this dispersed phase was determined using a FEI Helios Nano lab 600i), as shown in Figure 3. The dispersed phase contained Fe, Mo, Cr, and V [20]. In addition, the level of Mo was close to that of Fe, while the Cr and V levels were lower. Therefore, the dispersed phase was defined as M6C. However, because the dispersed phase observed in this work was small, the obtained chemical composition may not be accurate, and the determined Fe content of the dispersed phase may be slightly higher than the actual value. In this study, the Fe and Mo particles were dominant, while the Cr and V particles were very lower. Therefore, the particles can be still be defined as M6C according to [20]. In conclusion, for M50NiL steel, coarse-grained samples without particles and fine-grained samples with particles can be obtained by different heat treatments.

### 2.2. Methods

First, both the fine- and coarse-grained samples were machined into small cylinders with a diameter of 8 mm and a length of 12 mm. Then, isothermal deformation tests were performed on a Gleeble-3500 thermo-mechanical simulator at 950–1100 °C and a strain rate of 0.01–1 s^−1^. The exact experimental procedure is as follows. First, two specimens with different initial states were prepared by heating to 1100 °C for 5 min and to 1150 °C for 30 min, respectively, at a constant rate of 20 °C/s. Next, all specimens were cooled to compression temperature at 10 °C/s for 10 s, and then were compressed until reaching a total true strain value of 1 at strain rates of 0.01, 0.1, and 1 s^−1^; this was followed by immediate quenching in water. The stress–strain curves obtained during the hot deformation were automatically saved in a computer program. After completion of the hot deformation process, the deformed samples were cut along the longitudinal direction using an electric spark cutting machine. Subsequently, the surface of the samples was ground and polished. Thereafter, the samples were electrolytically etched in a supersaturated solution of oxalic acid to reveal the microstructure of the M50NiL steel. For microstructure characterization, the maximum deformation zone of the samples was observed using optical microscopy, scanning electron microscopy equipped with a backscattered electron (SEM–BSE) detector, and transmission electron microscopy (TEM).

## 3. Results and Discussions

### 3.1. Flow Stress Behavior of M50NiL Steel

**Figure 4:**
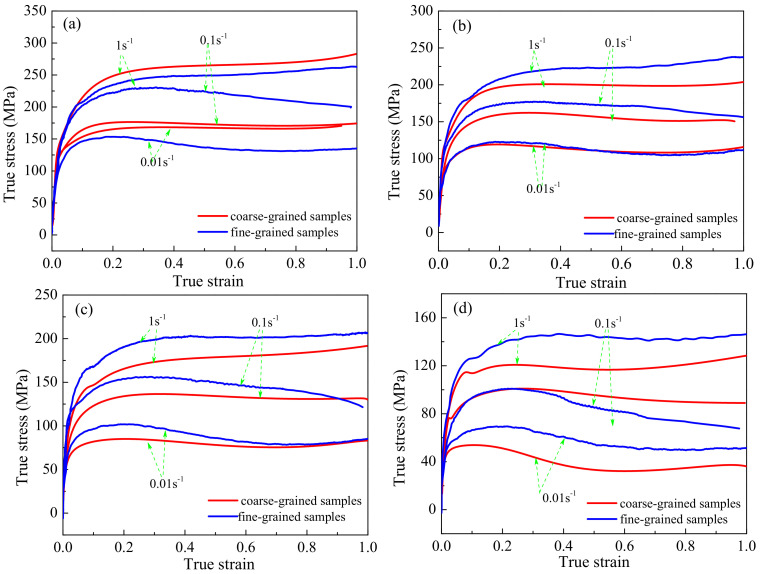
True stress–strain curves of the fine- and coarse-grained samples at different temperatures: (**a**) 950 °C; (**b**) 1000 °C; (**c**) 1050 °C; (**d**) 1100 °C.

The true stress–strain curves of the fine- and coarse-grained specimens under a deformation temperature of 950–1100 °C and a strain rate of 0.01–1 s^−1^ are presented in Figure 4. According to the true stress–strain curves, both samples exhibited their peak stress value at the initial deformation stage, which then decreased or reached a steady-state with increasing strain.

In general, stress gradually increases at the initial stage, and then reaches a steady-state without significant softening and clear peaks, which is considered a characteristic of dynamic recovery (DRV) or continuous dynamic recrystallization (CDRX). After reaching a peak stress, the flow stress decreases, which is considered a characteristic of discontinuous dynamic recrystallization (DDRX). It can be seen that in the true stress–strain curves, the DDRX characteristics occupied a dominant part at a higher deformation temperature and lower strain rate (Figure 4d), which indicates that the DDRX behavior was apparent at higher deformation temperatures and lower strain rates. This behavior can be attributed to the fact that, at higher temperatures, the available energy for the mobility of atoms is higher, and more point defeats and dislocations can be produced [21]. In addition, the critical resolved shear stress decreases at high temperatures [22]. Moreover, lowering the strain rate can weaken the work-hardening effect and extend the recrystallization time.

Figure 4 shows that the coarse-grained samples exhibited higher flow stress levels than the fine-grained ones at a deformation temperature of 950 °C. At the deformation temperatures investigated in this study, the Hall–Petch relationship broke down due to grain boundary sliding [23]. That is to say, under these deformation conditions, the flow stress increased with increasing grain size. In addition, the increase in temperature decreased the effectiveness of grain size on flow stress alternation [10,23]. Consequently, only at the deformation temperature of 950 °C was the flow stress of the coarse-grained sample higher than that of the fine-grained one.

**Figure 5:**
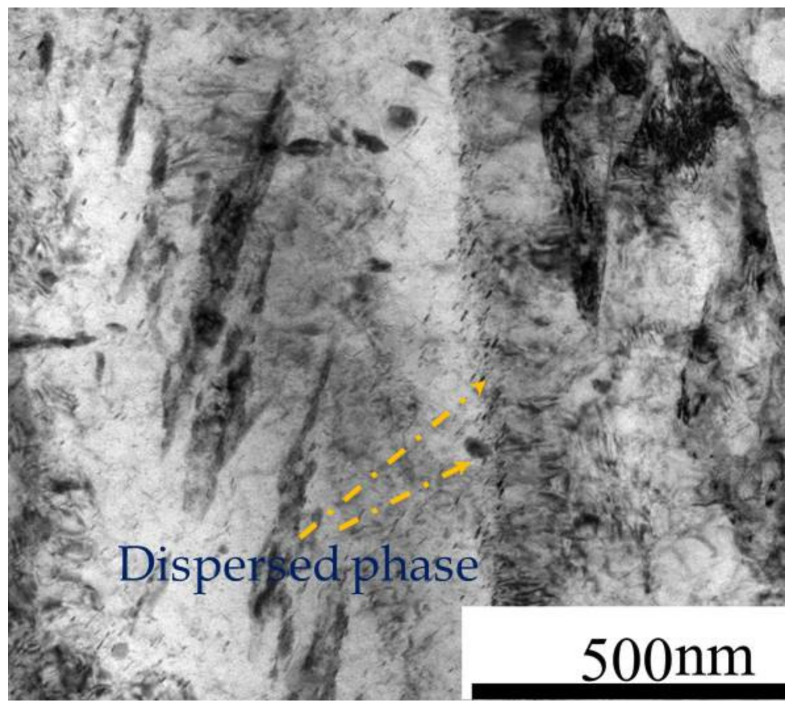
Transmission electron microscopy (TEM) of the fine-grained samples of M50NiL steel.

It can be seen in Figure 4 that the flow stress of the fine-grained samples was higher than that of the coarse-grained samples at a deformation temperature of 1000–1100 °C. In general, due to Zener pinning, dispersion is of great significance to the microstructure evolution of alloys [15]. The pinning effect is related to the size, volume fraction, and spatial distribution of the dispersed phase. Typically, the fine dispersed phase (<1 μm) will pin the grain boundary, thus hindering the nucleation and growth of recrystallization. In this study, the particle size of the dispersed phase was 0.27–0.32 μm; thus, the grain boundary was pinned by Zener pinning. In Figure 5, it can be seen that a large number of dislocations were generated around the dispersed phase, which is attributed to the pinning effect of the dispersed phase. In general, true stress–strain curves can be considered as a macroscopic reflection of the microstructural evolution of materials. The interaction between the dispersed phase and dynamic recrystallization has different effects on the flow stress level. More specifically, when the dispersed phase inhibits dynamic recrystallization, the flow stress usually significantly increases [24]. Hence, under most deformation conditions, the flow stress of fine-grained samples is higher than that of coarse-grained samples, which can be attributed to the pinning effect of the dispersed phase.

### 3.2. Deformation Energy of M50NiL Steel

As mentioned above, both the temperature and strain rate have considerable effects on flow stress. The effect of both temperature and strain rate can be incorporated in the Z parameter, as shown in Equation (1) [18]:

(1) $$Z=\;\dot{\varepsilon }\exp\left(\frac{Q}{\mathrm{RT}}\right)=\left\{\begin{array}{l} A^{\prime \prime }\sigma^{n^{\prime }}\\ A^{\prime }\exp\left(\beta \sigma \right)\\ A{\left[\sin h\left(\alpha \sigma \right)\right]}^{n} \end{array}\right.$$

where T is the absolute temperature (K), $\dot{\varepsilon }$ is the strain rate (s^−1^), σ is the flow stress (MPa), Q is the activation energy (J·mol^−1^), R is the universal gas constant (8.314 J·mol^−1^·K^−1^), and n, $n^{\prime }$, A, $A^{\prime }$, $A^{\prime \prime }$, β, and α are the material constants. The value of α can be calculated according to ${\alpha =\beta /n}^{\prime }$.

In this study, the peak stress, σ_p_, was used to calculate the material constant, and then a logarithm was applied to both sides of Equation (1):

(2) $$\mathrm{lnZ}=\ln\;\dot{\varepsilon }\;+\frac{Q}{\mathrm{RT}}=\left\{\begin{array}{l} {\mathrm{lnA}}^{\prime \prime }{+n^{\prime }\ln\sigma }_{p}\\ {\mathrm{lnA}}^{\prime }{+\beta \sigma }_{p}\\ \mathrm{lnA}+\mathrm{nln}\left[\sin h\left({\alpha \sigma }_{p}\right)\right] \end{array}\right.$$

Then, Equation (2) was partially differentiated at constant T, leading to Equation (3):

(3) $$\begin{array}{l} n^{\prime }={\left[\partial \ln\;\dot{\varepsilon }/\partial {\ln\sigma }_{p}\right]}_{T}\\ \beta ={\left[\partial \ln\;\dot{\varepsilon }/\partial \sigma_{p}\right]}_{T}\\ n={\left[\partial \ln\;\dot{\varepsilon }/\partial \ln\;\mathrm{lnsinh}\left({\alpha \sigma }_{p}\right)\right]}_{T} \end{array}$$

**Figure 6:**
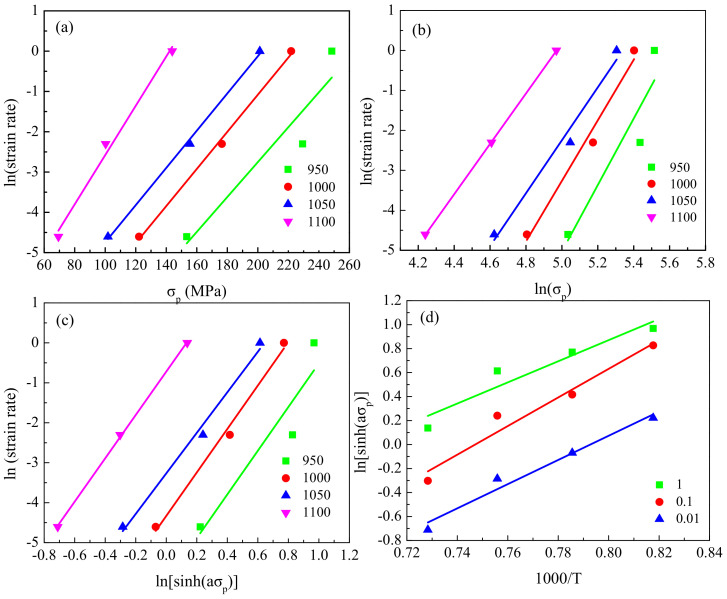
Relationships between: (**a**) $\ln\dot{\varepsilon }$ and σ_p_; (**b**) $\ln\dot{\varepsilon }$ and lnσ_p_; (**c**) $\ln\dot{\varepsilon }$ and lnsinh (ασ_p_); (**d**) lnsinh (ασ_p_) and 1000/T, for the fine-grained specimens.

**Figure 7:**
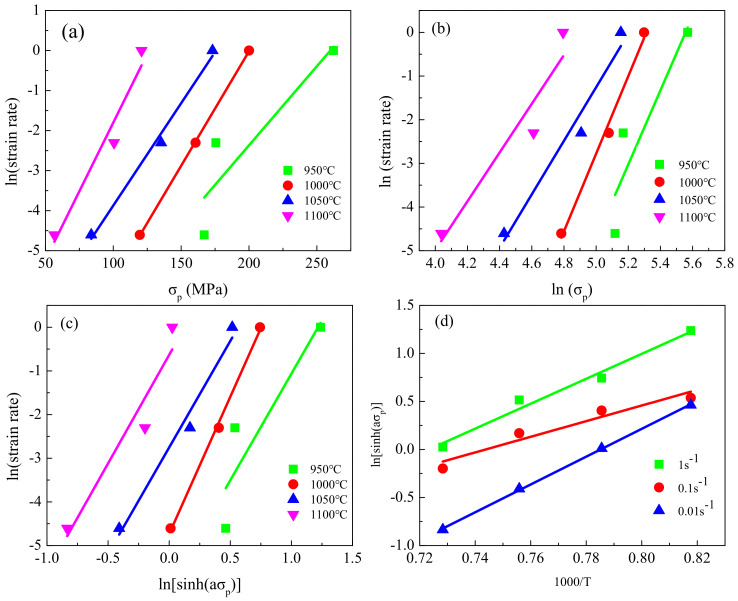
Relationships between: (**a**) $\ln\dot{\varepsilon }$ and σ_p_; (**b**) $\ln\dot{\varepsilon }$ and lnσ_p_; (**c**) $\ln\dot{\varepsilon }$ and lnsinh(ασ_p_); (**d**) lnsinh (ασ_p_) and 1000/T, for the coarse-grained specimens.

By substituting the corresponding experimental data in Equation (2) and by applying linear regression, the relationship between strain rate and stress was obtained (Figure 6 and Figure 7). As per Equation (3), the values of $n^{\prime }$ and β were derived from the slope of ln$\dot{\varepsilon }$ versus lnσ_p_ and the slope of ln$\dot{\varepsilon }$ versus σ_p_, respectively. The values of $n^{\prime }$ and β were, respectively, determined as 0.054 and 7.27 for the coarse-grained specimens and 0.049 and 7.21 for the fine-grained specimens. Then, α was calculated as 0.007 for both types of specimens.

For constant $\dot{\varepsilon }$, partial differentiation of Equation (2) yields Equation (4):

(4) $$Q=\mathrm{Rn}{\left[\frac{\partial \ln\left[\sin h\left({\alpha \sigma }_{p}\right)\right]}{\partial \left(1/T\right)}\right]}_{\dot{\varepsilon }}=\mathrm{Rns}$$

where s can be determined from the slope of lnsinh(ασ_p_) versus 1/T, and n can be determined from the slope of ln $\dot{\varepsilon }$ versus lnsinh(ασ_p_). As seen in Figure 6 and Figure 7, the values of s and n were, respectively, determined as 11.3 and 5.25 for the coarse-grained specimens and 10.28 and 5.35 for the fine-grained specimens. Finally, the activation energy for the coarse- and fine-grained specimens was determined to be 493.85 KJ·mol^−1^ and 457.16 KJ·mol^−1^, respectively. Moreover, the obtained activation energy for the coarse- and fine-grained samples was similar to the 481.4 KJ·mol^−1^ obtained by Ji [16] for M50NiL steel.

The activation energy, Q, is an important material parameter that serves as an indicator of the degree of deformation difficulty during hot deformation [25]. In general, the activation energy value for hot deformation is affected by factors such as the chemical composition, initial microstructure, alloy type, and test conditions [26]. In Zr- and Cu-based alloys [10,27], the deformation activation of coarse-grained specimens has been found to be higher than that of fine-grained specimens. However, in austenitic stainless steel [28], when the grain size is smaller than 60 μm, the deformation activation energy increases with increasing grain size, while the opposite phenomenon occurs when the grain size is larger than 60 μm. Some studies have reported that the dispersed phase makes the operation of the deformation and recovery mechanisms more difficult, which leads to an increase in the activation energy [29,30]. In this work, the activation energy of the fine-grained samples was close to that of the coarse-grained samples, which can be attributed to the joint effect of the grain size and the dispersed phase.

### 3.3. Critical Strain

**Figure 8:**
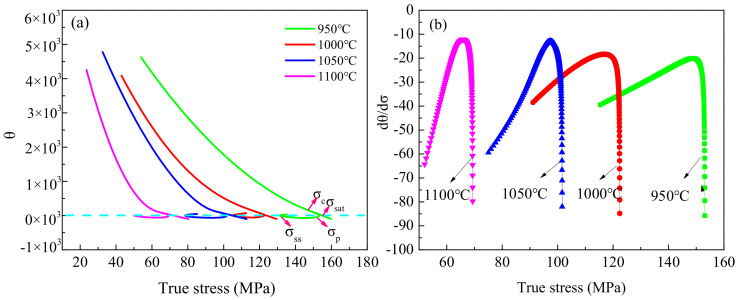
(**a**) θ-σ curves and (**b**) dθ/dσ-σ curves of the fine-grained specimens at a strain rate of 0.01 s^−1^ for different deformation temperatures.

**Figure 9:**
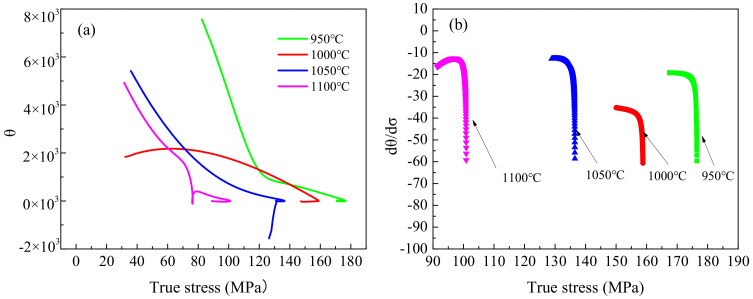
(**a**) θ-σ curves and (**b**) dθ/dσ-σ curves of the coarse-grained specimens at a strain rate of 0.01 s^−1^ for different deformation temperatures.

The critical strain of dynamic recrystallization constitutes one of the important indicators in the analysis of dynamic recrystallization behavior, since it can determine the strain required for the initiation of dynamic recrystallization [31]. The critical strain can be determined by metallurgical observations; however, this requires considerable effort and time. In [32,33], it was demonstrated that when dynamic recrystallization occurs, the curves of the hardening rate θ=∂σ∂ε versus true stress $\left(\theta \right)$ exhibit an inflection point. The inflection point in the θ-σ curves corresponds to the maximum point of the ∂θ∂σ-σ curves. The θ-σ and ∂θ∂σ-σ curves derived based on the obtained true stress–strain curves are shown in Figure 8 and Figure 9. Subsequently, the critical strains under different hot deformation conditions were determined.

**Figure 10:**
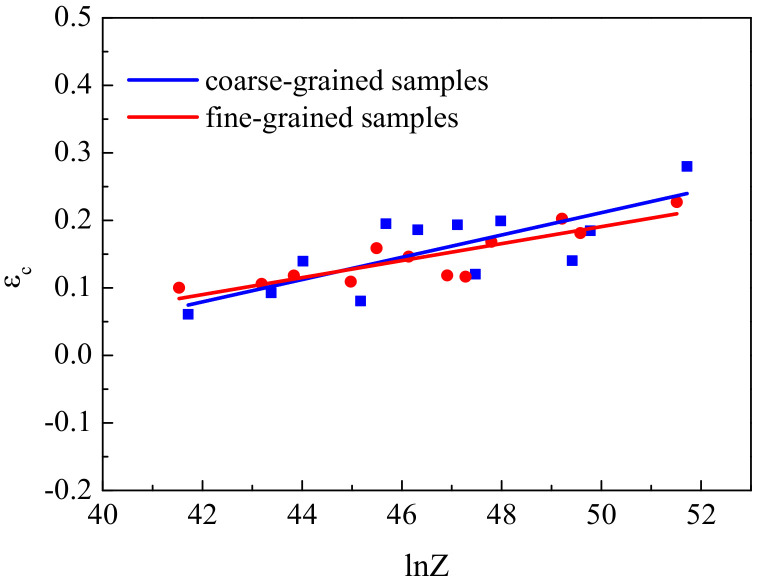
Relationship between ε_c_ and lnZ for the fine- and coarse-grained samples.

As mentioned above, the dynamic recrystallization behavior is affected by the temperature and strain rate. Therefore, the critical strain can be expressed by the Z parameter as:

ε_c_ = AZ^k^ (5)

where A and k are material constants. By substituting the corresponding critical strain and Z parameter into Equation (5), the relationship between the critical strain and Z parameter can be obtained (Figure 10).

It can be seen in Figure 10 that under most deformation conditions, the critical strain of the fine-grained specimens was lower than that of the coarse-grained ones. Chen [34] also reported that the critical strain decreases with decreasing initial grain size in 3003 aluminum alloy. Therefore, the recrystallization of fine grains is more likely to occur. It is generally accepted that the DRX process is a nucleation and growth phenomenon. The nucleation of strain-free grains occurs at high-energy sites, such as the grain boundaries and other defect regions such as dislocation clusters. However, with increasing grain size, the volume fraction of the grain boundaries decreases, and the accumulation of dislocation density is expected to be low. Consequently, the reduction in DRX kinetics with increasing initial grain size is an obvious consequence of the lack of nucleation sites [10].

### 3.4. Evolution of Dynamic Recrystallization Under Different Hot Deformation Conditions

**Figure 11:**
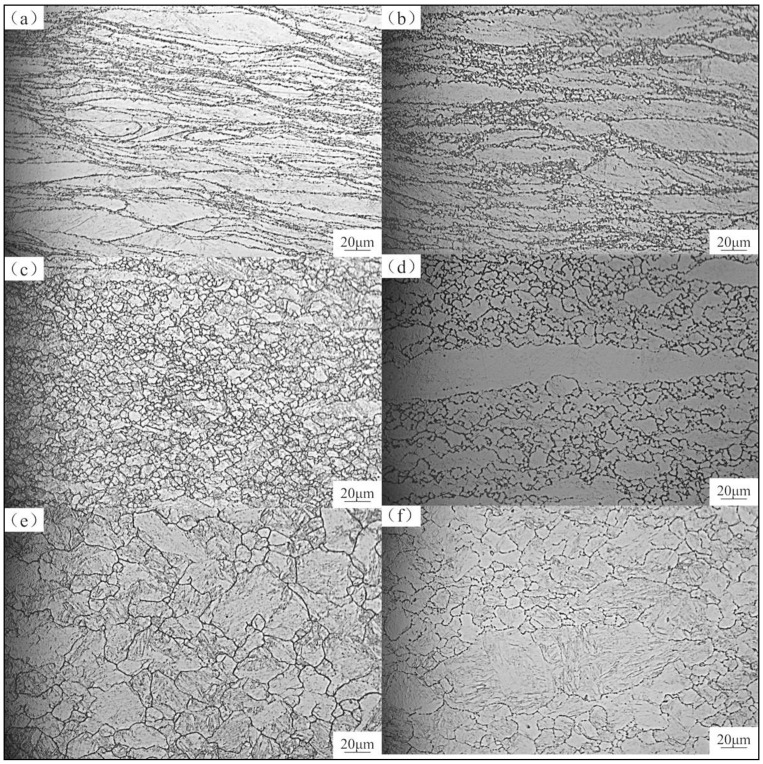
Optical micrographs of the fine- and coarse-grained specimens at different deformation conditions: (**a**,**c**,**e**) fine-grained samples; (**b**,**d**,**f**) coarse-grained samples; (**a**,**b**) high Z; (**c**,**d**) intermediate Z; (**e**,**f**) low Z.

To investigate the microstructure evolution during hot deformation, optical microscopy images of both samples under different hot deformation conditions were captured, as shown in Figure 11. Similar to the observations of the true stress–strain curves in Figure 4, dynamic recrystallization occurred during hot deformation and was affected by the hot deformation temperature and strain rate.

In both samples investigated here, a typical necklace structure was observed (Figure 11a,c). With decreasing strain rate and increasing deformation temperature (with decreasing Z), the necklace structure gradually disappeared and more fractions of new grains appeared. This demonstrates that dynamic recrystallization tends to occur at higher deformation temperatures and lower strain rates. This is because higher temperatures can provide more energy for the mobility of dislocations, and lower strain rates can provide more time for dynamic recrystallization.

In Figure 11b,e, it can be seen that, at intermediate Z, the dynamic recrystallization in the fine-grained samples was completed, and the dynamically recrystallized grain size (DRX grain size) in the fine-grained samples was smaller than that in the coarse-grained ones. This is because dynamic recrystallization behavior is more likely to occur in fine-grained samples, and the second phase in the fine-grained samples can pin the grain boundaries and hinder the growth of recrystallized grains. In addition, immediately after completion of the recrystallization in both the coarse- and fine-grained samples, the DRX grain size of the coarse grains was 9.19 μm, while that of the fine grains was 5.54 μm. Consequently, the M50NiL steel microstructure with fine grains and dispersed phase is more conducive to obtaining a uniform and fine microstructure and better mechanical properties.

## 4. Conclusions

The aim of the present study was to assess the effect of the initial microstructure and hot deformation parameters on the evolution of dynamic recrystallization of M50NiL steel. To this end, hot deformation experiments were performed at temperatures between 950 and 1100 °C and strain rates between 0.01 and 1 s^−1^ on a Gleeble-3500 testing device. Based on the results and analysis, the following conclusions can be drawn:

1. The true stress–strain curve can be considered a macroscopic reflection of the changes in the microstructure of a material. Under most deformation conditions, the flow stress of the fine-grained specimens was higher than that of the coarse-grained specimens, which is attributed to the pinning effect of the fine-grain dispersion on the grain boundary.
2. The critical strain and deformation activation energy of the fine-grained samples were lower than those of the coarse-grained samples. It was proven that a microstructure with fine grains is beneficial to the dynamic recrystallization of M50NiL steel.
3. The original microstructure with fine grains and dispersed phase is more favorable for the hot deformation of M50NiL steel. This is due to the fact that the dispersed phase can pin the grain boundaries and hinder the growth of recrystallized grains. Consequently, a more uniform and finer microstructure can be obtained after recrystallization.

## Figures and Tables

**Table 1:** Composition (wt.%) of M50NiL steel.

C	Si	Mn	P	S	Cr	Ni	V	Mo
0.13	0.19	0.29	0.005	0.002	4.14	3.4	1.24	4.07
